# Modulatory effects of acupuncture on raphe nucleus‐related brain circuits in patients with chronic neck pain: A randomized neuroimaging trial

**DOI:** 10.1111/cns.14335

**Published:** 2023-07-05

**Authors:** Xiao Wang, Xixiu Ni, Xu Ouyang, Yutong Zhang, Tao Xu, Linjia Wang, Wenchuan Qi, Mingsheng Sun, Qian Zeng, Ziwen Wang, Huaqiang Liao, Xiaoyu Gao, Dehua Li, Ling Zhao

**Affiliations:** ^1^ Acupuncture and Tuina School, Chengdu University of Traditional Chinese Medicine Chengdu Sichuan China; ^2^ Acupuncture and Moxibustion Clinical Medical Research Center of Sichuan Province Chengdu University of Traditional Chinese Medicine Chengdu Sichuan China; ^3^ Hospital of Chengdu University of Traditional Chinese Medicine Chengdu Sichuan China

**Keywords:** fMRI, neck pain, raphe nucleus, serotonergic

## Abstract

**Objective:**

Acupuncture has shown promise in treating neck pain. Clinical trials have shown mixed results, possibly due to heterogeneous methodologies and the lack of knowledge regarding underlying brain circuit mechanism of action. In this study, we investigated the specific contribution of the serotonergic system in treating neck pain, and the specific brain circuits involved.

**Methods:**

A total of 99 patients with chronic neck pain (CNP) were randomized to receive true acupuncture (TA) or sham acupuncture (SA) 3 times weekly for 4 weeks. Patients with CNP in each group were assessed for primary outcomes by measuring the Visual Analog Scale (VAS) and the duration of each attack; secondary outcomes were measured using the Neck Disability Index (NDI), Northwick Park Neck Pain Questionnaire (NPQ), McGill Pain Questionnaire (MPQ), Self‐rating Anxiety Scale (SAS), Self‐rating Depression Scale (SDS) and the 12‐item Short Form Quality Life Scale (SF‐12); levels of functional circuits connectivity were assessed using resting‐state functional magnetic resonance imaging in the dorsal (DR) and median (MR) raphe nucleus, before and after undergoing acupuncture.

**Results:**

Patients receiving TA showed more extensive symptom improvement compared with SA. Regarding the primary outcomes, changes observed in the TA group were as follows: VAS = 16.9 mm (*p* < 0.001) and the duration of each attack = 4.30 h (*p* < 0.001); changes in the SA group: VAS = 5.41 mm (*p* = 0.138) and the duration of each attack = 2.06 h (*p* = 0.058). Regarding the secondary outcomes, changes in the TA group: NDI = 7.99 (*p* < 0.001), NPQ = 10.82 (*p* < 0.001), MPQ = 4.23 (*p* < 0.001), SAS = 5.82 (*p* < 0.001), SDS = 3.67 (*p* = 0.003), and SF‐12 = 3.04 (*p* < 0.001); changes in the SA group: NDI = 2.97 (*p* = 0.138), NPQ = 5.24 (*p* = 0.035) and MPQ = 2.90 (*p* = 0.039), SAS = 1.48 (*p* = 0.433), SDS = 2.39 (*p* = 0.244), and SF‐12 = 2.19 (*p* = 0.038). The modulatory effect of TA exhibited increased functional connectivity (FC) between the DR and thalamus, between the MR and parahippocampal gyrus, amygdala, and insula, with decreased FC between the DR and lingual gyrus and middle frontal gyrus, between the MR and middle frontal gyrus. Furthermore, changes in the DR‐related circuit were specifically associated with the intensity and duration of pain, and the MR‐related circuit was correlated with the quality of life with CNP.

**Conclusion:**

These results demonstrated the effectiveness of TA in treating neck pain and suggested that it regulates CNP by reconfiguring the function of the raphe nucleus‐related serotonergic system.

## Graphical abstract

True acupuncture treatment showed significant treatment effects by regulating the raphe nucleus‐related serotonergic circuits in chronic neck pain.

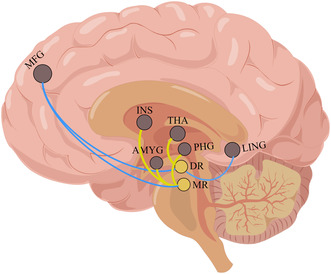

## 1 INTRODUCTION

Neck pain, with an annual prevalence rate exceeding 30%, is the fourth leading cause of disability.^1^ Most episodes of acute neck pain will resolve with or without treatment; however, nearly 50% of individuals continue to experience some degree of pain or frequent recurrences.^2^ Drug therapy, including paracetamol, non‐steroidal drugs, anti‐inflammatory drugs, and opioids, is a common treatment for acute and chronic neck pain (CNP).^3^ However, the pharmacological management for low back and neck pain costs over 100 billion dollars in the United States alone^4^, ^5^ and often causes non‐negligible side effects including fatigue, nausea, headache, and increased pain.^6^ Moreover, long‐term use of some medications can significantly increase the risk of drug dependence.^7^ Because of these limitations create an imperative for evidence‐based therapies with elucidated mechanisms a priority.

In addition to medication, diverse treatments exist for neck pain including physical therapy and complementary and alternative medicine therapies. Although acupuncture is widely used, its effectiveness remains controversial. Several trials with small sample sizes have shown that true acupuncture (TA) is more effective than sham acupuncture (SA; shallow puncture or needling at non‐acupoint locations) for relieving neck pain by modifying the perception of pain or altering physiological functions, that is, pain control for treating certain diseases or dysfunctions.^8^ However, other trials have shown that acupuncture reduces neck pain and produces a statistically, but not clinically, significant effect compared with that of placebo.^8^ These beneficial effects may be due to non‐specific or specific.^7^ Although many systematic reviews and meta‐analyses of CNP have been conducted, most of them have been inconclusive, and this has led to confusion in clinical policy and practice.^9^ The inconsistency in these findings may have resulted from variations in the design characteristics (such as the choice of SA) and the lack of clear longitudinal neuroimaging evidence.

Many studies have reported that acupuncture stimulation can relieve chronic pain by modulating the trigeminal spino‐thalamo‐cortical circuit,^10^, ^11^, ^12^, ^13^ which is a key network in the regulation of pain,^10^ and has a high degree of congruence with the serotonin‐acting regions.^14^, ^15^ The raphe nucleus, an important node in this circuit, is the major source of serotonin, which is crucial for endogenous control including pain inhibition, negative affect, in vivo passive coping, and catastrophizing.^16^, ^17^, ^18^, ^19^ Recent studies have focused on acupuncture for treating chronic pain via this descending pain regulation loop. Gao et al^20^ found that acupuncture improves pain intensity by enhancing the functional connectivity (FC) of the dorsal raphe (DR) nucleus in knee osteoarthritis with chronic pain. Liang et al^11^ reported that acupuncture relieves knee osteoarthritis knee pain by modulating the FC between the ventrolateral periaqueductal gray and thalamus. However, little is known about whether and how acupuncture modulates CNP via this circuit.

Functional magnetic resonance imaging (fMRI) is an effective tool for detecting the functional integrity of specific brain circuits in vivo.^21^, ^22^, ^23^ Han et al^24^ used the raphe nucleus as seeds and found that the dysconnectivity between it and the subcortical serotonin‐related regions contributes to altered salience network in schizophrenia. Similarly, increased FC between the raphe nucleus and periaqueductal gray has been reported in chronic migraine.^25^ Using resting‐state FC, Li et al. reported the abnormal dynamic activity of the striatal‐sensorimotor circuit in patients with benign epilepsy.^26^ Therefore, fMRI is suitable for exploring the raphe nucleus‐related serotonin circuit in CNP.

In this trial, we compared the clinical outcomes of TA with those of SA, and investigated how acupuncture improves the clinical outcomes of patients with CNP by regulating the raphe nucleus‐related serotonergic system. We hypothesized that TA could better reduce pain intensity, duration and pain‐related disability and improve the quality of life of patients with CNP. Moreover, we proposed a new perspective, in which TA was effective by reconfiguring the function of the serotonin system in CNP.

## 2 METHODS

We collected the clinical and fMRI data of patients with CNP from the Hospital of Chengdu University of Traditional Chinese Medicine (TCM), where the participants were recruited from the acupuncture and orthopedic clinics. The research protocol was submitted to the Chinese Clinical Trial Registry with the identifier (ChiCTR1800017718) and was reviewed by the Sichuan Regional Ethics Review Committee on Traditional Chinese Medicine (ethical approval number: 2018KL‐056). The recruitment process started since August 2018 and continued until December 2019.

### 2.1 Participants

We included patients with CNP, whose criteria were based on the guidelines developed by the Orthopedic Section of the American Physical Therapy Association.^27^ The inclusion criteria were as follows: (1) 18–65 years old men or women with neck pain and discomfort or limited cervical motion as the main symptoms; (2) right‐hand dominance; (3) pain score of ≥4 cm on the Visual Analog Scale (VAS) for 5 of 7 days (range, 0–10 cm); (4) more than 3 months duration of disease; and (5) informed consent signed by patients. Patients with any of the following conditions were excluded: (1) macroscopic T2‐visible brain lesions on the MRI scans; (2) accompanied by other serious organic lesions, including malignant neoplasms, tuberculosis, fracture, or osteomyelitis; (3) complications associated with serious primary diseases, including cardiovascular, cerebrovascular, liver, kidney, and hematopoietic systems; (4) mental disorders and other mental disorders that could not be matched with the questionnaire (a score of self‐rating anxiety scale [SAS] or self‐rating depression scale [SDS] > 72); (5) bleeding tendencies, allergies, and skin diseases; pregnancy, lactation, or fertility issues within the past 6 months; (6) contraindications such as metals in the body; and (7) participation in other simultaneous clinical trials.

### 2.2 Study design

**FIGURE 1:**
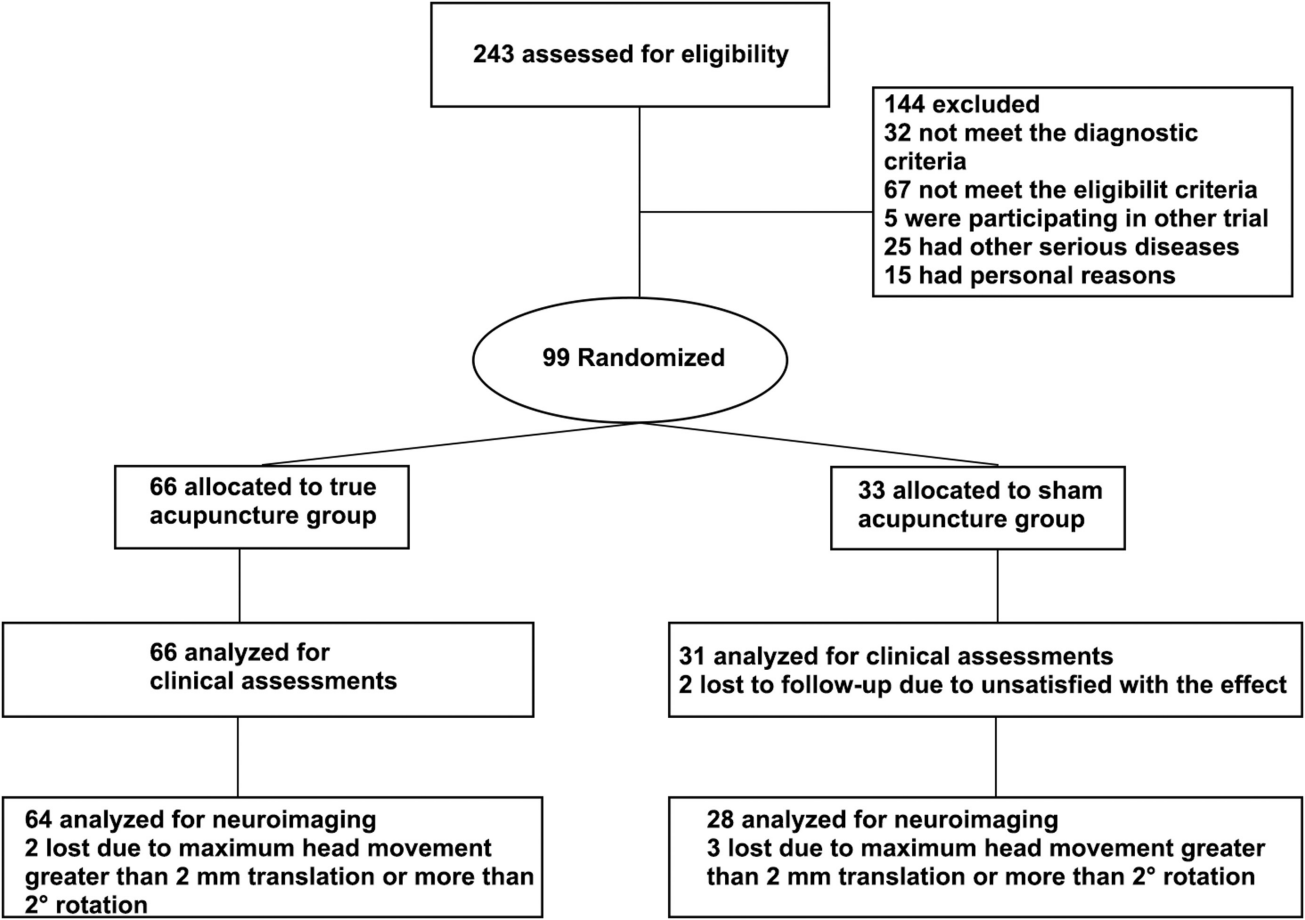
Study flowchart.

In this trial, the patients experiencing CNP were observed for 6 weeks, of which the first 2 weeks of the run‐in period checked whether patients with CNP were eligible and willing to participate. A total of 99 patients with CNP were randomized to receive TA or SA with a 2:1 ratio^28^, ^29^, ^30^, ^31^ (TA = 66; SA = 33) by using a random number table (Figure 1). An independent staff generated the random number and grouping scheme using the SPSS software (version 20.0; IBM Corp.) to set the block length and number of segments to nine according to the block and the number of segments to 11 according to the block randomization principle. The TA and SA groups were blinded to their group assignments and were treated in a closed unit. Acupuncturists could not be blinded to the treatment assignments because of the nature of the interventions; thus, they were not engaged in the evaluation of outcomes or data analysis. Other researchers, including outcome assessors, data collectors, and statisticians, were blinded to the treatment allocation.

### 2.3 Intervention

**FIGURE 2:**
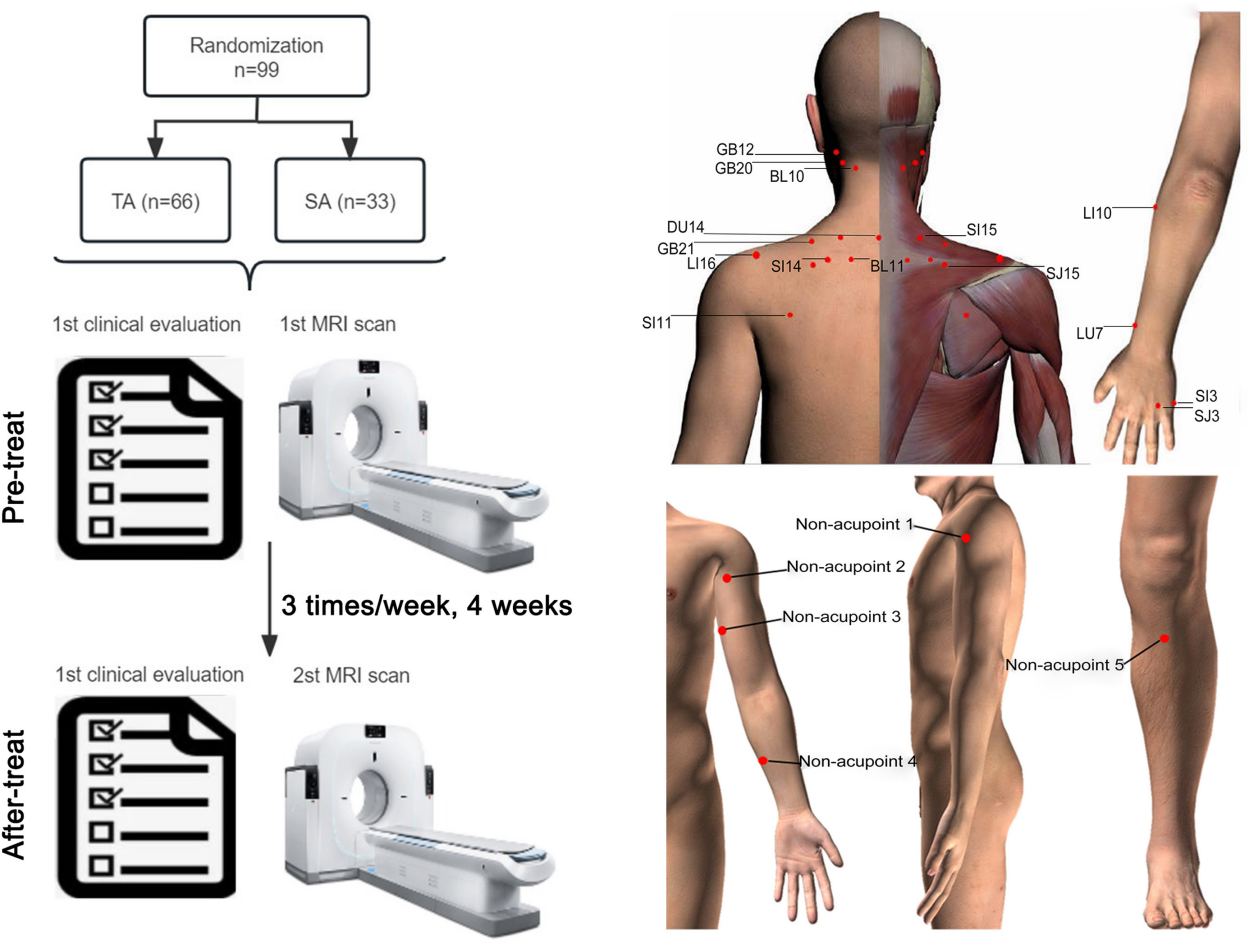
Experimental design and acupoint/non‐acupoint location. SA, sham acupuncture; TA, true acupuncture. Non‐acupoint 1, midpoint between the brachial arch and acromial arch; non‐acupoint 2, medial edge of the upper arm, the joint of the deltoid muscle, and biceps brachii muscle; non‐acupoint 3, midpoint between the elbow tip and axilla; non‐acupoint 4, midpoint between the medial epicondyle of the humerus and the ulnar end of the wrist stripe; and non‐acupoint 5, tibial front.

Acupuncture therapy was administered to both groups in turn by two specialized acupuncturists with at least 5 years of training and 3 years of experience. The acupoints and non‐acupoints used in this study are shown in Figure 2. After detecting individualized tenderness threshold at 15 acupoints, five sensitive acupuncture points with the largest absolute value of changes in the pressure pain threshold were selected as the treatment acupoints in the TA group, including the Tianliao point (SJ15), Jianwaishu point (SI14), Jianzhongshu point (SI15), Dazhu point (BL11), and Jugu point (LI16). The 15 acupoints were determined by data mined from the literature and expert consensus on the treatment of CNP, including Jianjing (GB21), Jianzhongshu (SI15), Wangu (GB12), Fengchi (GB20), Tianzhu (BL10), Dazhui (DU14), Dazhu (BL11), Jianwaishu (SI14), Tianliao (SJ15), Jugu (LI16), Tianzong (SI11), Shousanli (LI10), Lieque (LU7), Zhongzhu (SJ3), and Houxi (SI3).^32^, ^33^ The 5 non‐acupoints used in the SA group were selected based on previous studies.^34^, ^35^, ^36^ We have previously published a clinical study protocol related to the acupoint selection and treatment.^36^

All acupoints were punctured using single‐use stainless steel filiform needles (Hwato Needles, Sino‐foreign Joint Venture Suzhou *Hua Tuo* Medical Instruments Co.), which were 25–40 mm in length and 0.25–0.30 mm in diameter. In the TA group, the depths of the inserted needles varied but were approximately 0.5–1.5 cm. Rotating or lifting–thrusting manipulation was performed for *Deqi* sensation (a sensation of soreness, numbness, distention, or radiation that indicated effective needling) after needle insertion. In the SA group, shallow acupuncture with a depth of 2 mm was performed at the five non‐acupoints, without manual stimulation to yield the *Deqi* sensation.^37^ Both groups received acupuncture treatment 3 times weekly for 4 weeks with a total of 12 sessions. Each session lasted for 30 min. None of the patients were allowed to receive any sustained‐release or prophylactic analgesics. For intolerable neck pain, patients were allowed to receive analgesic medications (such as non‐steroidal anti‐inflammatory drugs) or effective analgesic medications, which they considered as rescue medications. The details are documented in a Case Report Form.

### 2.4 Clinical assessments

Changes in the VAS score (with 0 representing no pain and 10 representing the worst pain imaginable) and the duration of each attack from baseline to 4 weeks of treatment were set as the primary outcomes to assess the severity of the chronic pain. Secondary outcomes included the Neck Disability Index (NDI), Northwick Park Neck Pain Questionnaire (NPQ), and McGill Pain Questionnaire (MPQ) for holistic pain measurement. The SDS/SAS and 12‐item Short Form Quality Life Scale (SF‐12) were further used as secondary outcomes to evaluate the pain‐related impairment of emotion and quality of life. The pre‐ and post‐treatment values for each group were compared using paired *t* tests.

### 2.5 MRI data acquisition

fMRI images were acquired using a 3.0 T MRI scanner (GE, Discovery MR750) in the MRI Center of the Hospital of the Chengdu University of TCM. All participants were instructed to rest with their eyes closed, not to think of anything in particular, and not to fall asleep during the scan. The functional images were collected transversely using a gradient‐recalled echo‐planar imaging pulse sequence with the following settings: TR/TE = 2000/30 ms, flip angle = 90°, 40 slices, slice thickness = 5 mm, 64 × 64 matrix, field of view = 250 × 250 mm^2^, interslice gap = 0 mm, and voxel size = 3.75 × 3.75 × 5 mm^3^. The scan lasted for 7 min for each subject, and 210 functional volumes were obtained.

### 2.6 Data analysis

#### 2.6.1 Clinical data analysis

The sample size, which was calculated using the NQuery Advisor software (version 4.0; Statistical Solutions) with a 2‐sided significance level of 5% and a power of 90%, included 80 participants.^38^ The estimated lost‐to‐follow‐up rate was 15%.^39^ Thus, we enrolled 99 participants in the two groups (66 in the TA group and 33 in the SA group).

All statistical evaluations were performed using SPSS 22.0 (SPSS Inc.). The normality distribution of the data was assessed using the visual inspection of histograms and the Shapiro–Wilk test. Data that did not follow a normal/Gaussian distribution were analyzed using a non‐parametric test. We used the following statistical tests: the chi‐squared test, Mann–Whitney *U* test, and Kruskal–Wallis *H* test. Statistical analysis was performed using a two‐tailed test, and the significance level was set at 5%.

#### 2.6.2 Raphe nucleus seed‐based FC analysis

Resting‐state fMRI images were preprocessed using the data processing and analysis of brain imaging toolbox. The first 5 volumes were discarded to avoid the non‐equilibrium effects of magnetization, and slice timing and realignment correction were performed for the remaining images. Any participant with maximum head movement >2.0 mm translation or >2.0° rotation was not included. In this step, two patients in the TA group and three patients in the SA group were excluded from the following analyses. Data were further normalized to the EPI template (resampled voxel size: 3 × 3 × 3 mm^3^). Several covariates including the Fristion 24 motion parameters, cerebrospinal fluid, and white matter signals were regressed as nuisance variables to reduce spurious variance. No global signal regression was performed to avoid introducing distortion into the time series data.^40^ Detrending and band‐pass filtering (0.01–0.08 Hz) were then conducted. Finally, since the resting‐state activity is sensitive to minor head movements, we calculated the mean frame‐wise displacement (FD) to further determine the comparability of head movement among groups. “Bad” time points (FD > 0.5 mm), as well as their one‐back and two‐forward time points, were scrubbed and interpolated by spline interpolation.^41^

As described in previous positron emission tomography (PET) and fMRI studies, the raphe nucleus is subdivided into the DR and median raphe (MR) nucleus.^42^, ^43^ The central coordinates of the seeds were obtained with a spherical radius of 4 mm. Accordingly, the ROIs were defined as follows: DR (0, −27, −9) and MR (0, −31, −21)^27^ (Figure 4). The voxel‐wise FC between the seeds and each voxel in the brain was then calculated using the Pearson's correlation and the correlation coefficients were converted to *Z*‐values using the Fisher's *r*‐to‐*z* transformation.

ancova analysis was used to assess the reconfiguration of the TA on the raphe nucleus‐related circuits with age and gender as covariates in CNP. Gaussian random field corrections (with voxel *p* < 0.005, cluster *p* < 0.05) were conducted for the multiple comparison correction.^29^ Correlation analysis was performed between the altered FC between the seed regions to the ROIs and the improvements in clinical symptoms in patients with CNP.

## 3 RESULTS

After 243 patients were screened, 99 with CNP aged 18–65 years were randomized, of whom 63 (64.9%) were women. Two patients in the TA group discontinued the study and three patients in the SA group discontinued the neuroimaging analyses because of a maximum head movement >2 mm translation or >2° rotation. Two patients in the SA group discontinued the clinical assessment because of unsatisfactory outcomes. Finally, 97 patients (66 in the TA group and 31 in the SA group) were included in the final clinical analysis, and 92 patients (64 in the TA group and 28 in the SA group) were included in the neuroimaging analysis (Figure 1). No significant difference in head movement was observed among the groups (TA‐before treatment: 0.07 ± 0.03; TA‐after treatment: 0.11 ± 0.06; SA‐before treatment: 0.13 ± 0.04; SA‐after treatment: 0.09 ± 0.07; FD mean ± SD, *p* = 0.37).

### 3.1 Patient characteristics

The baseline demographic characteristics of all patients are summarized in Table 1. These values were comparable between the TA and SA groups (*p* > 0.05). Two patients (2.1%) in the SA group did not undergo follow‐up because of unsatisfactory outcomes.

**TABLE 1:** Participant demographic and baseline characteristics.

Items	TA (*N* = 66)	SA (*N* = 31)	*p*‐value
Age, mean (SD), years	46.57 (13.26)	47.64 (14.83)	0.741^b^
Gender (male/female)	22/44	12/19	0.605^a^
Weight, mean (SD), kg	57.16 (9.25)	60.51 (9.76)	0.132^b^
Education, mean (SD), years	12.93 (4.02)	13.07 (3.55)	0.870^b^
Duration of illness, mean (SD), months	83.14 (60.49)	96.96 (94.75)	0.575^b^
VAS scores, mm, mean (SD)	58.14 (13.2)	54.78 (13.3)	0.363^b^
Duration of each attack, mean (SD), h	9.03 (6.60)	8.15 (6.04)	0.532^b^
Pain medication use (y/n)	2/64	2/29	0.429^a^
NPQ, mean (SD)	36.76 (15.27)	34.90 (12.22)	0.556^b^
NDI, mean (SD)	29.99 (13.73)	27.89 (13.82)	0.491^b^
MPQ, mean (SD)	20.01 (8.04)	19.22 (8.34)	0.660^b^
SAS, mean (SD)	45.83 (9.88)	45.83 (9.88)	0.366^b^
SDS, mean (SD)	41.92 (9.62)	44.22 (13.56)	0.407^b^
SF‐12, mean (SD)	31.40 (6.45)	31.77 (6.11)	0.794^b^

*Note*: *p* ^b^‐value was obtained by χ^2^ two‐tailed test. *p* ^a^‐value was obtained by two‐sample *t* test.

Abbreviations: MPQ, McGill Pain Questionnaire; NDI, Neck Disability Index; NPQ, Northwick Park Neck Pain Questionnaire; SA, sham acupuncture; SAS, Self‐rating Anxiety Scale; SD, standard deviation; SDS, Self‐rating Depression Scale; SF‐12, 12‐item Short Form Quality Life Scale; TA, true acupuncture; VAS, Visual Analog Scale.

### 3.2 Clinical outcomes

**FIGURE 3:**
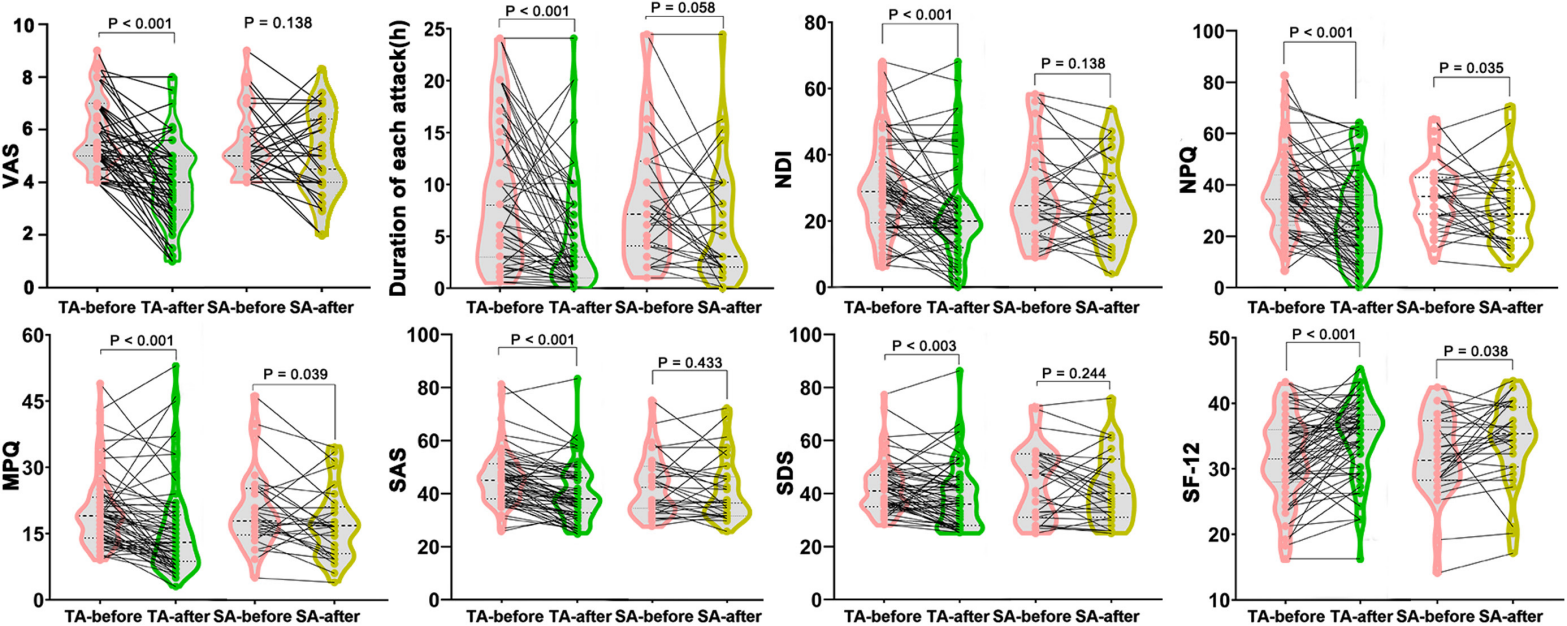
Clinical outcomes in TA and SA groups, respectively. MPQ, McGill Pain Questionnaire; NDI, Neck Disability Index; NPQ, Northwick Park Neck Pain Questionnaire; SA, sham acupuncture; SAS, Self‐rating Anxiety Scale; SDS, Self‐rating Depression Scale; SF‐12, 12‐item Short Form Quality Life Scale; TA, true acupuncture; VAS, Visual Analog Scale.

The clinical outcomes conformed to a normal distribution and are presented in both the scatter and distribution plots shown in Figure 3.

#### 3.2.1 Primary outcomes

Table 2 and Figure 3 show the clinical outcomes of the TA and SA groups. The results were analyzed to determine the changes that occurred from baseline to the end of the 4‐week treatment period. Regarding the primary outcomes, the VAS scores significantly decreased in the TA group (16.9 mm, 95% CI: 12.15–21.61 mm, *p* < 0.001) and did not in the SA group (5.41 mm, 95% CI: 1.85–12.68 mm, *p* = 0.138), and the duration of each attack was significantly reduced in the TA group (4.30 h, 95% CI: 2.70–5.90 h, *p* < 0.001) and was not in the SA group (2.06 h, 95% CI: 0.07–4.19 h, *p* = 0.058) after 4 weeks of treatment, with a significant difference between the two groups (*p* < 0.05).

**TABLE 2:** Clinical outcomes during the study.

Items	TA (*N* = 66)	SA (*N* = 31)	*p* ^T, G^
Use of acute medicine (y/n)			
Baseline	2/64	1/30	0.959
Treatment 1–4 weeks	0/66	0/31	–
*p* ^a^	0.154^a^	0.313^a^	
VAS scores, mm			
Baseline (mean ± SD)	58.14 (13.2)	54.78 (13.3)	*p* ^T^ = 0.000
Treatment‐baseline (difference, 95% CI)	16.9 (12.15–21.61)	5.41 (1.85–12.68)	*p* ^T*G^ = 0.008
*p* ^b^	<0.001^b^	0.138^b^	*p* ^G^ = 0.327
Duration of each attack, h			
Baseline (mean ± SD)	9.03 (6.60)	8.15 (6.04)	*p* ^T^ = 0.000
Treatment‐baseline (difference, 95% CI)	4.30 (2.70–5.90)	2.06 (0.07–4.19)	*p* ^T*G^ = 0.105
*p* ^b^	<0.001^b^	0.058^b^	*p* ^G^ = 0.835
NDI			
Baseline (mean ± SD)	29.99 (13.73)	27.89 (13.82)	*p* ^T^ = 0.000
Treatment‐baseline (difference, 95% CI)	7.99 (4.92–11.06)	2.97 (1.01–6.94)	*p* ^T*G^ = 0.880
*p* ^b^	<0.001^b^	0.138^b^	*p* ^G^ = 0.491
NPQ			
Baseline (mean ± SD)	36.76 (15.27)	34.90 (12.22)	*p* ^T^ = 0.000
Treatment‐baseline (difference, 95% CI)	10.82 (7.18–14.46)	5.24 (0.04–10.09)	*p* ^T*G^ = 0.077
*p* ^b^	<0.001^b^	0.035^b^	*p* ^G^ = 0.748
MPQ			
Baseline (mean ± SD)	20.01 (8.04)	19.22 (8.34)	*p* ^T^ = 0.000
Treatment‐baseline (difference, 95% CI)	4.23 (2.17–6.28)	2.90 (0.15–5.65)	*p* ^T*G^ = 0.445
*p* ^b^	<0.001^b^	0.039^b^	*p* ^G^ = 0.942
SAS			
Baseline (mean ± SD)	45.83 (9.88)	43.58 (11.49)	*p* ^T^ = 0.000
Treatment‐baseline (difference, 95% CI)	5.82 (3.90–7.73)	1.48 (2.33–5.29)	*p* ^T*G^ = 0.024
*p* ^b^	<0.001^b^	0.433^b^	*p* ^G^ = 0.968
SDS			
Baseline (mean ± SD)	41.92 (9.62)	44.22 (13.56)	*p* ^T^ = 0.007
Treatment‐baseline (difference, 95% CI)	3.67 (1.31–6.03)	2.39 (1.72–6.49)	*p* ^T*G^ = 0.563
*p* ^b^	0.003^b^	0.244^b^	*p* ^G^ = 0.204
SF‐12			
Baseline (mean ± SD)	31.40 (6.45)	31.77 (6.11)	*p* ^T^ = 0.000
Treatment‐baseline (difference, 95% CI)	3.04 (1.61–4.46)	2.19 (0.13–4.26)	*p* ^T*G^ = 0.503
*p* ^b^	<0.001^b^	0.038^b^	*p* ^G^ = 0.963

*Note*: To make the improvement in clinical scores easier to understand, the difference values (treatment minus baseline) were taken as absolute values. *p* ^a^ value was obtained by χ^2^ two‐tailed test. *p* ^b^ value was obtained by paired‐*t* test within‐group. *p* ^T^ values for comparison between different time points. *p* ^T*G^ based on Time*Group interaction. *p* ^G^ based on comparison between different groups.

Abbreviations: MPQ, McGill Pain Questionnaire; NDI, Neck Disability Index; NPQ, Northwick Park Neck Pain Questionnaire; SA, sham acupuncture; SAS, Self‐rating Anxiety Scale; SD, standard deviation; SDS, Self‐rating Depression Scale; SF‐12, 12‐item Short Form Quality Life Scale; TA, true acupuncture; VAS, Visual Analog Scale.

#### 3.2.2 Secondary outcomes

Regarding the secondary outcomes, the NDI (7.99, 95% CI: 4.92–11.06, *p* < 0.001), NPQ (10.82, 95% CI: 7.18–14.46, *p* < 0.001), and MPQ (4.23, 95% CI: 2.17–6.28, *p* < 0.001) significantly decreased in the TA group. The NPQ (5.24, 95% CI: 0.04–10.09, *p* = 0.035) and MPQ (2.90, 95% CI: 0.15–5.65, *p* = 0.039) decreased in the SA group.

Regarding the further secondary outcomes, the SAS (5.82, 95% CI: 3.90–7.73, *p* < 0.001) and SDS (3.67, 95% CI: 1.31–6.03, *p* = 0.003) significantly decreased; and the SF‐12 (3.04, 95% CI: 1.61–4.46, *p* < 0.001) increased in the TA group. The SF‐12 (2.19, 95% CI: 0.13–4.26, *p* = 0.038) increased in the SA group.

### 3.3 Neuroimaging outcomes

**FIGURE 4:**
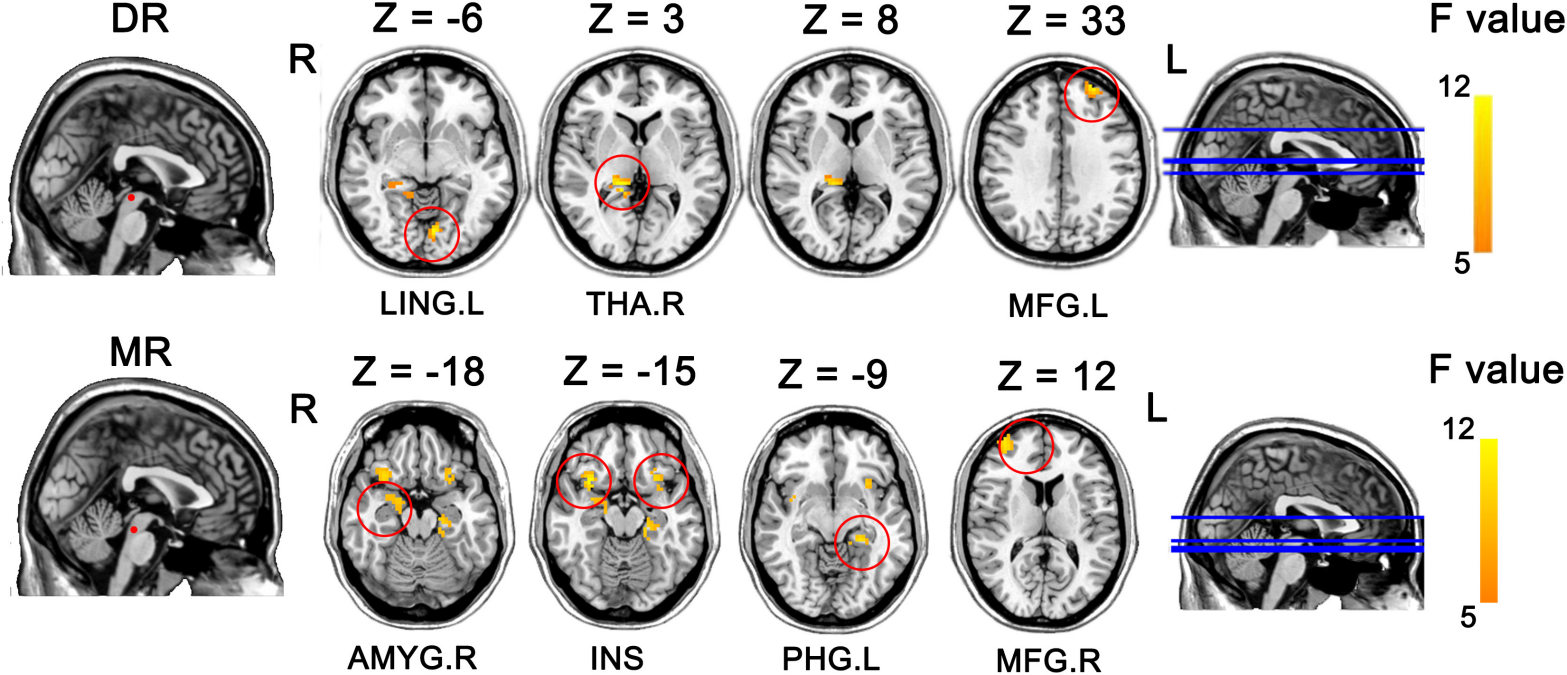
ancova analyses revealed the interaction effect of time and group factors. AMYG, amygdala; DR, dorsal raphe nucleus; INS, insula; L, left; LING, lingual gyrus; MFG, middle frontal gyrus; MR, median raphe nucleus; PHG, parahippocampal gyrus; R, right; THA, thalamus.

Considering the impact of head movement on imaging results, the neuroimaging analyses included 92 patients with CNP (TA = 64; SA = 28) who did not differ in gender, age, or years of education. As shown in Figure 4 and Table 3, with the DR as the seed, ancova analyses showed a significant interaction effect (time and group factors), mainly located in the left lingual gyrus, right thalamus, and left middle frontal gyrus (*p* < 0.05). With the MR as the seed, ancova analyses showed a significant interaction effect (time and group factors), mainly located in the right amygdala, bilateral insula, left parahippocampal gyrus, and right middle frontal gyrus (*p* < 0.05). A significant main effect of group factors was mainly located in the left lingual gyrus, right thalamus, and left middle frontal gyrus with the DR as the seed, and in the right amygdala with the MR as the seed. The main effects of time were mainly located in the right thalamus, bilateral insula, and left parahippocampal gyrus.

**TABLE 3:** Neuroimaging outcomes obtained by ancova analyses with time and group as factors.

Brain area	TA (*N* = 64)	SA (*N* = 28)	*P* ^T, G^
DR_LING.L			
Baseline (mean ± SD)	0.13 (0.10)	0.06 (0.011)	*p* ^T^ = 0.073
Treatment‐baseline (difference, 95% CI)	0.06 (0.01 to 0.121)	−0.03 (−0.09 to −0.026)	*p* ^T*G^ = 0.0013
			*p* ^G^ = 0.027
DR_THA.R			
Baseline (mean ± SD)	0.18 (0.11)	0.23 (0.11)	*p* ^T^ = 0.000
Treatment‐baseline (difference, 95% CI)	−0.05 (−0.11 to 0.005)	0.08 (0.02 to 0.142)	*p* ^T*G^ = 0.0009
			*p* ^G^ = 0.008
DR_MFG.L			
Baseline (mean ± SD)	0.12 (0.07)	0.09 (0.08)	*p* ^T^ = 0.220
Treatment‐baseline (difference, 95% CI)	0.022 (−0.01 to 0.05)	−0.06 (−0.10 to −0.02)	*p* ^T*G^ = 0.0026
			*p* ^G^ = 0.009
MR_AMYG.R			
Baseline (mean ± SD)	0.09 (0.09)	0.12 (0.09)	*p* ^T^ = 0.059
Treatment‐baseline (difference, 95% CI)	−0.04 (−0.09 to 0.001)	−0.045 (0.01 to 0.09)	*p* ^T*G^ = 0.0059
			*p* ^G^ = 0.015
MR_INS.L			
Baseline (mean ± SD)	0.08 (0.09)	0.14 (0.1)	*p* ^T^ = 0.012
Treatment‐baseline (difference, 95% CI)	−0.07 (−0.13 to −0.01)	0.07 (−0.01 to 0.12)	*p* ^T*G^ = 0.0042
			*p* ^G^ = 0.102
MR_INS.R			
Baseline (mean ± SD)	0.07 (0.09)	0.15 (0.11)	*p* ^T^ = 0.012
Treatment‐baseline (difference, 95% CI)	−0.05 (−0.10−−0.01)	‐0.01 (−0.003 to 0.09)	*p* ^T*G^ = 0.0009
			*p* ^G^ = 0.098
MR_PHG.L			
Baseline (mean ± SD)	0.13 (0.07)	0.19 (0.08)	*p* ^T^ = 0.004
Treatment‐baseline (difference, 95% CI)	−0.06 (−0.10 to −0.02)	0.04 (−0.01 to 0.09)	*p* ^T*G^ = 0.0019
			*p* ^G^ = 0.114
MR_MFG.R			
Baseline (mean ± SD)	0.12 (0.10)	0.05 (0.08)	*p* ^T^ = 0.095
Treatment‐baseline (difference, 95% CI)	0.04 (−0.01 to 0.09)	−0.06 (−0.10 to −0.01)	*p* ^T*G^ = 0.0022
			*p* ^G^ = 0.264

*Note*: *p* ^T^ values for comparison between different time points. *p* ^T*G^ based on Time*Group interaction. *p* ^G^ based on the comparison between different groups.

Abbreviations: AMYG, amygdala; DR, dorsal raphe nucleus; INS, insula; L, left; LING, lingual gyrus; MFG, middle frontal gyrus; MR, median raphe nucleus; PHG, parahippocampal gyrus; R, right; SA, sham acupuncture; TA, true acupuncture; THA, thalamus.

**FIGURE 5:**
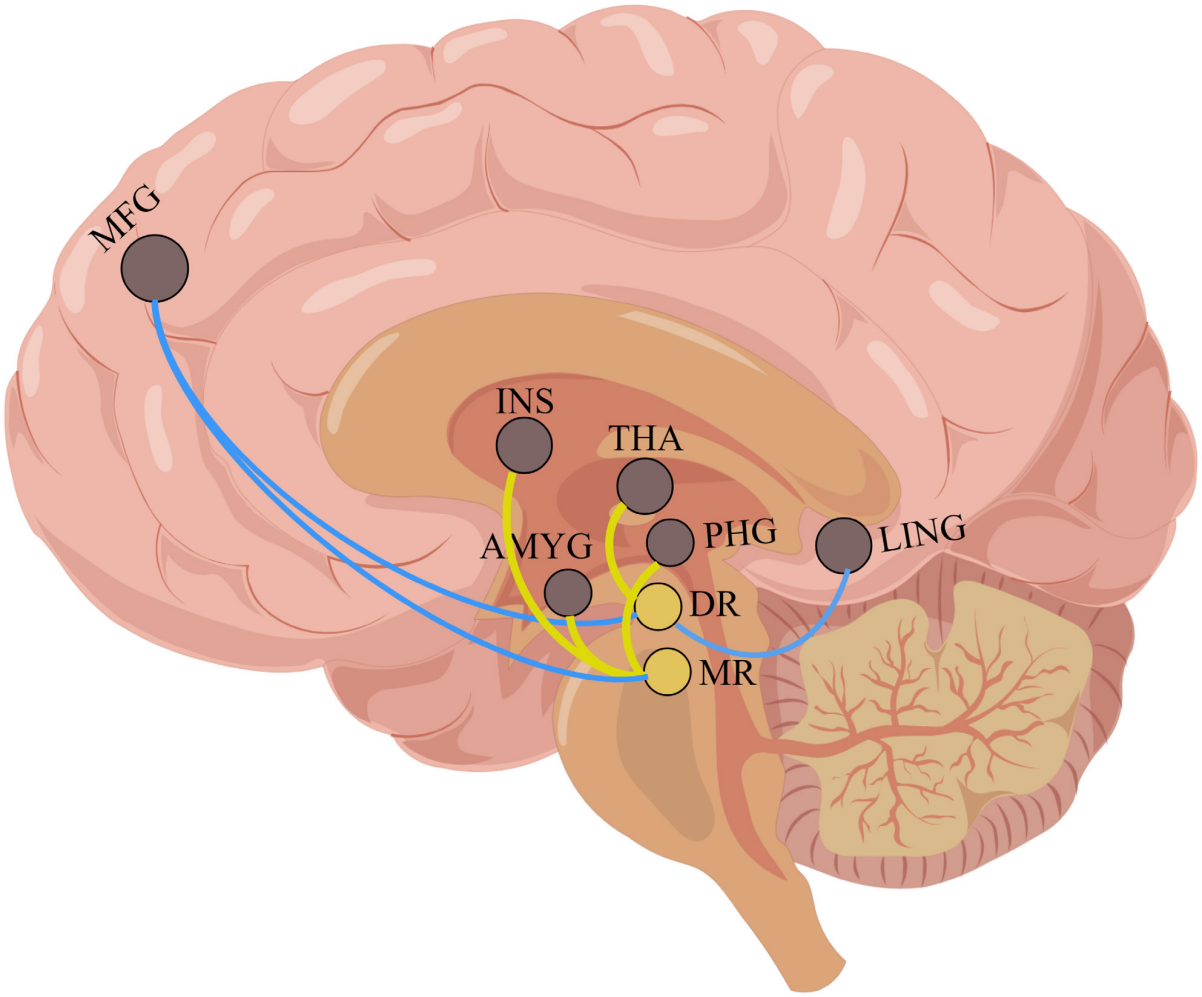
The reconfiguration of TA on the raphe nucleus‐related serotonergic circuits in CNP. The warmer yellow color represents the higher *F* value in the ancova analyses. The yellow color line denotes TA increased the FC between the regions in CNP. The blue color line denotes TA decreased FC between the regions in CNP. AMYG, amygdala; DR, dorsal raphe nucleus; INS, insula; LING, lingual gyrus; MFG, middle frontal gyrus; MR, median raphe nucleus; PHG, parahippocampal gyrus; THA, thalamus.

Post hoc analyses revealed reconfiguration of the TA in the FC of the raphe nucleus subdivision in CNP (Figure 5).

### 3.4 Correlation with the clinical improvements in the TA group

**FIGURE 6:**
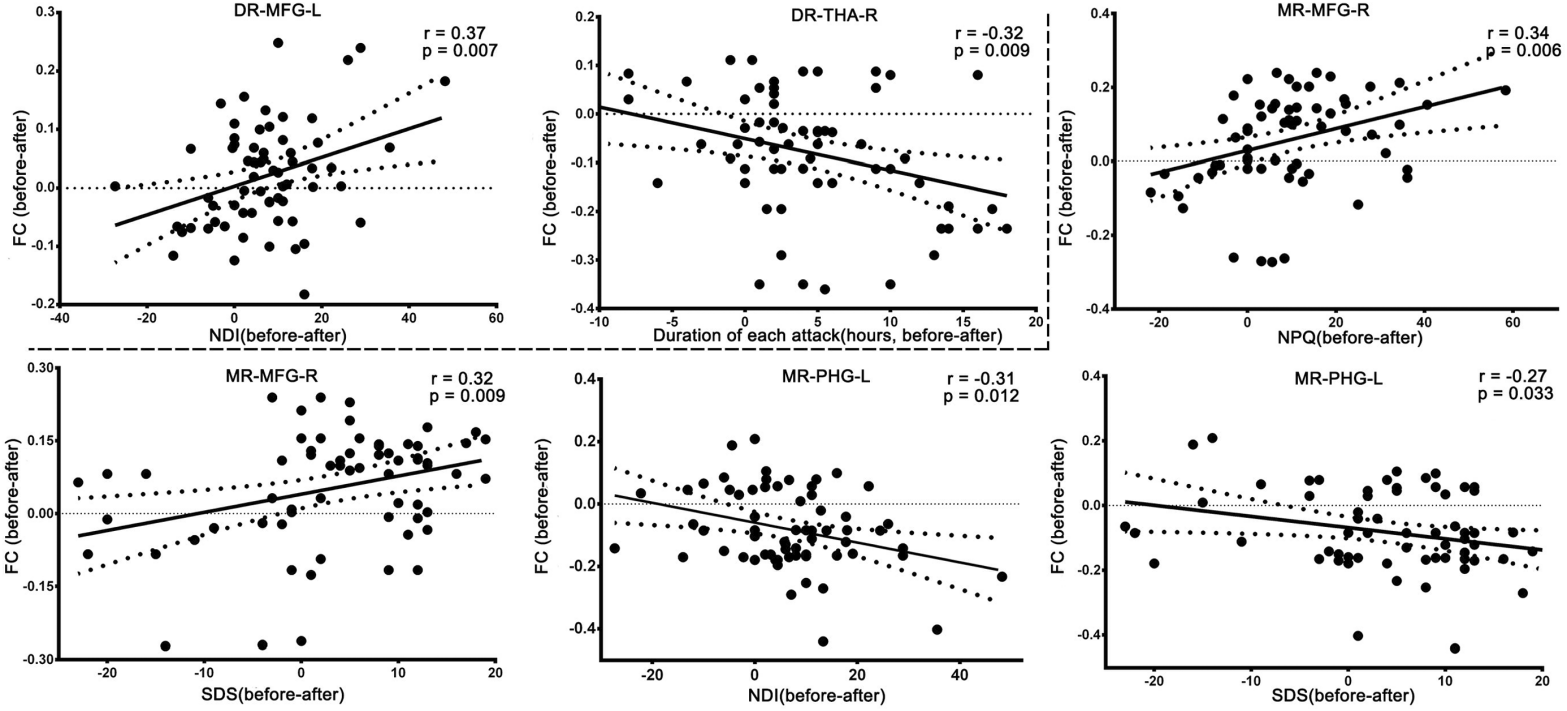
Correlation with the clinical improvements in the TA group. DR, dorsal raphe nucleus; FC, functional connectivity; L, left; MFG, middle frontal gyrus; MFG, middle frontal gyrus; MR, median raphe nucleus; NDI, Neck Disability Index; NPQ, Northwick Park Neck Pain Questionnaire; PHG, parahippocampal gyrus; R, right; SDS, Self‐rating Depression Scale; THA, thalamus.

As shown in Figure 6, changes in FC between the DR and left middle frontal gyrus were positively correlated with the NDI improvements in the TA group (*r* = 0.37, *p* = 0.007), while those between the DR and right thalamus were inversely correlated with the duration of each attack improvement in the TA group (*r* = −0.32, *p* = 0.009). Moreover, changes in FC between the MR and right middle frontal gyrus were positively correlated with improvements in the NPQ (*r* = 0.34, *p* = 0.006) and SDS (*r* = 0.32, *p* = 0.009) scores, while those between the MR and parahippocampal gyrus were inversely correlated with improvements in the NDI (*r* = −0.31, *p* = 0.012) and SDS (*r* = −0.27, *p* = 0.033) scores in the TA group.

### 3.5 Medication uses

No patient reported pain medication usage during the 4‐week treatment period in this study. However, four patients used a sticking plaster on the neck for pain relief (two in the TA group and two in the SA group).

### 3.6 Patients safety

All 99 patients were monitored for safety and tolerability. Four patients in TA and three patients in the SA group developed mild bruising around the acupoints/non‐acupoints, all of which were back to normal after 1 week of follow‐up.

## 4 DISCUSSION

TA has demonstrated significant and clinically relevant benefits for CNP, as it reduces the pain intensity and duration of each attack compared with SA. Moreover, improvements in pain‐related emotional impairment and quality of life were observed. Furthermore, SA had a weak statistically significant effect in controlling pain‐related disability measures, including the NPQ and MPQ, after 4 weeks of treatment. Thus, acupuncture should be considered a treatment option for neck pain. Moreover, we found that the modulation of the TA mainly included the DR‐thalamus‐frontal gyrus and MR‐limbic‐frontal gyrus circuits. The former was specifically associated with the primary outcomes (intensity and duration of pain), whereas the latter was associated with the secondary outcomes. To the best of our knowledge, this study is the first randomized neuroimaging trial to combine clinical and neuroimaging analyses to test the efficacy of TA and explore its underlying neuroimaging mechanisms in CNP. Rigorous paired control groups were used to assess both the clinical and neuroimaging outcomes, providing valuable insights into the role of raphe nucleus‐related circuits as neuroimaging markers in acupuncture treatment for CNP.

First, we found that TA was effective in reducing the intensity of neck pain and duration of each attack, and it was more effective than SA in reducing the VAS scores in CNP. These findings are congruent with those of previous clinical trials^44^ and demonstrated the effectiveness of acupoint TA in relieving chronic pain.^45^ In addition, TA showed effectiveness in improving secondary clinical outcomes including the NDI, NPQ, MPQ, SAS, and SDS, as well as, in increasing the SF‐12 scores. Some studies have shown that acupuncture can effectively reduce pain and improve function in patients with CNP, supporting its appropriateness and effectiveness in treating chronic pain.^46^, ^47^ However, a study using SA found that although the VAS scores in both groups were significantly reduced after therapy, there is no significant difference between groups was detected.^48^ Similar to our findings, most clinical outcomes were significantly improved in the TA group compared with those in the SA group, except for the VAS and SAS scores; this may be attributed to the placebo effect of SA. The control intervention method we selected was non‐acupoint shallow acupuncture, to explore the specificity of acupoints. Previous studies have suggested that acupuncture analgesia may work through diffuse harmful inhibitory controls, which are responses to a painful stimulus that suppresses pain from another stimulus (pain‐inhibiting‐pain effect).^49^ However, another study showed that the effect could be explained by improving tactile sensitivity due to primary somatosensory cortical neuroplasticity in specific body structures, which could be induced by penetrating the sham control.^50^ Moreover, needling can enhance patients' confidence in receiving acupuncture, indicating that non‐acupoint acupuncture has psychological analgesic effects similar to those of TA.^8^ Moreover, the study demonstrated that the placebo effect of acupuncture was definite and showed high variability among different types of sham controls and that the placebo effects were influenced by the depth of needle insertion and puncture location.^8^ Therefore, the placebo effect can be avoided to a large extent by using a non‐acupoint shallow puncture. Nevertheless, currently, there is still no consensus on the selection of control/sham acupuncture interventions, indicating the need for further search into different neuroimaging mechanisms.

To explore the modulation on the serotonergic system via TA in the treatment of CNP, we selected important source nucleus of serotonin in the raphe nucleus, that is, the DR and MR according to previous PET and fMRI studies. We found that TA modulated the functional connection between the DR and thalamocortical networks by reducing the FC between the DR and the lingual gyrus and middle frontal gyrus, but increasing the FC between the DR and thalamus. Consistent results have been reported for other chronic pain, such as chronic migraine^13^ and chronic low back pain.^51^ The DR, thalamus, and middle frontal cortex play central roles in pain processing. The thalamus integrates the pain stimulus and sends it to the high brain processing area (mainly located in the frontal cortex), which further converts the stimulus into a perceptual signal; this perceptual signal is further used to downregulate the pain (including in the subcortex and DR).^52^ Moreover, previous studies have demonstrated that the FC between the DR and frontal cortex/thalamus is the key target area of acupuncture in chronic pain.^53^, ^54^, ^55^ The modulation of these abnormalities by acupuncture contributes to pain processing, such as pain sensation and pain perception.^52^, ^56^ These findings suggest that acupuncture relieves and treats CNP by regulating endogenous pain processing via the DR‐related circuit.

Moreover, we found that TA modulated the connections between the MR to limbic networks and the middle frontal gyrus by increasing the FC between the MR and the amygdala, bilateral insula, and parahippocampal gyrus and reducing the FC between the MR and the middle frontal gyrus. Interestingly, acupuncture enhanced the association between the MR and limbic regions but weakened the association between the MR and frontal cortex. The limbic regions, particularly the insula, are important components of the pain matrix.^57^ Baliki et al. proved that the insula includes circuits specific to pain perception and is generally linked to encoding the intensity or magnitude of sensory stimuli, such as painful stimuli.^58^, ^59^ The amygdala plays a key role in emotion processing and the emotion‐affective dimension of pain.^60^, ^61^ Coppieters et al^61^ found that the dysfunction of the amygdala could affect the link between cognitive‐affective and sensory modulation of pain in CNP. The parahippocampal gyrus, which is located at the junction between the hippocampus and the fusiform cortex, is involved in diverse classes of stimuli, tasks, environments, and pain perception.^62^ These results suggest that acupuncture may alleviate a wider range of pain dimensions, such as pain emotion and perception, in CNP through the FC of the MR.

Correlation analysis revealed that the modulation of acupuncture on the FC of the DR was significantly correlated with improvement in the NDI and the duration of each attack. The modulation of the FC of the MR by TA was significantly associated with improvements in the NPQ, SDS, and NDI. The current findings suggest that acupuncture can regulate the DR‐related circuit to alleviate and treat CNP, including the sensation and perception of pain. Moreover, acupuncture can reduce pain‐related emotion impairment mainly by regulating the MR‐related limbic circuit in CNP. Overall, the subdivision of the raphe nucleus may play a unique role in using acupuncture in treating CNP.

This study has some limitations. First, it was a single‐center trial, which might have introduced potential biases that influenced the generalizability of the results. However, quality is more easily controlled and internal authenticity is better guaranteed in single‐center trials than in multicenter trials. Second, acupuncture was performed by experienced acupuncturists who could not be blinded. Whether non‐acupuncture practices produce similar results remained unknown. Third, the intervention was an individualized treatment; therefore, the results did not clearly explain the relationship between left‐ and right‐side pain and brain circuits. In the future, we will conduct a subgroup analysis of unilateral and bilateral pain in patients with neck pain to assess the specific relationship between neck pain, acupuncture points, and different brain circuits.

## 5 CONCLUSION

In summary, TA showed superior treatment effects in CNP than those of SA. TA resulted in significant reductions in the intensity and duration and improvements in emotional well‐being and quality of life, rather than being merely a placebo. After TA treatment, the improvement in primary and secondary clinical outcomes was significantly associated with changes in raphe nucleus‐related serotonergic circuits in CNP. These findings demonstrate that acupuncture can be considered a treatment option for CNP and support the hypothesis that acupuncture could treat CNP by regulating the serotonergic circuits of the brain.

## AUTHOR CONTRIBUTIONS

Ling Zhao is the corresponding author. Xiao Wang, Xixiu Ni, and Xu Ouyang contributed equally to this article. Ling Zhao, Xixiu Ni, and Yutong Zhang carried out study protocol and design. Tao Xu, Linjia Wang, Wenchuan Qi, Mingsheng Sun, Qian Zeng, Ziwen Wang, Huaqiang Liao, Xiaoyu Gao, and Dehua Li were involved in acquisition of data. Xiao Wang and Xu Ouyang carried out analysis and interpretation of data. Xiao Wang, Xixiu Ni, and Xu Ouyang carried out drafting of the manuscript. Ling Zhao contributed to proofreading and revising the manuscript. All authors reviewed the manuscript.

## FUNDING INFORMATION

This work was supported by the National Natural Science Foundation of China (grant no. 81722050, 82204919, 81973962, 82274664), China Postdoctoral Science Foundation (grant no. 2022MD713681), the Department of Science and Technology of Sichuan Province (2021ZYD0103), and Innovation Team and Talents Cultivation Program of National Administration of Traditional Chinese Medicine (grant no. ZYYCXTD‐D‐202003).

## CONFLICT OF INTEREST STATEMENT

The authors have no conflicts of interest to declare.

## Data Availability

The data that support the findings of this study are available from the corresponding author upon reasonable request.

## References

[1] Cohen SP . Epidemiology, diagnosis, and treatment of neck pain. Mayo Clin Proc. 2015;90(2):284‐299. 25659245 10.1016/j.mayocp.2014.09.008

[2] Resnik L , Borgia M , Clark MA . The prevalence and impact of back and neck pain in veterans with upper limb amputation. Am J Phys Med Rehabil. 2021;100(11):1042‐1053. 33443850 10.1097/PHM.0000000000001694

[3] Suto G . Non‐steroidal anti‐inflammatory drugs for relieving pain in musculoskeletal disorders. Orv Hetil. 2019;160(22):855‐860. 31131611 10.1556/650.2019.31502

[4] Huang JF , Meng Z , Zheng XQ , et al. Real‐world evidence in prescription medication use among U.S. adults with neck pain. Pain Ther. 2020;9(2):637‐655. 32940899 10.1007/s40122-020-00193-1 PMC7648792

[5] Dieleman JL , Cao J , Chapin A , et al. US health care spending by payer and health condition, 1996‐2016. JAMA. 2020;323(9):863‐884. 32125402 10.1001/jama.2020.0734 PMC7054840

[6] Machado GC , Maher CG , Ferreira PH , et al. Efficacy and safety of paracetamol for spinal pain and osteoarthritis: systematic review and meta‐analysis of randomised placebo controlled trials. BMJ. 2015;350:h1225. 25828856 10.1136/bmj.h1225 PMC4381278

[7] D'Arcy Y . Managing pain in a patient who's drug‐dependent. Nursing. 2007;37(3):36‐40. quiz 41. 10.1097/01.nurse.0000261823.50672.26 17546781

[8] Zeng D , Yan XX , Deng HM , et al. Placebo response varies between different types of sham acupuncture: a randomized double‐blind trial in neck pain patients. Eur J Pain. 2022;26(5):1006‐1020. 35129852 10.1002/ejp.1924 PMC9305463

[9] Paley CA , Johnson MI . Acupuncture for the relief of chronic pain: a synthesis of systematic reviews. Medicina (Kaunas). 2019;56(1):6. 31878346 10.3390/medicina56010006 PMC7023333

[10] Mercer Lindsay N , Chen C , Gilam G , Mackey S , Scherrer G . Brain circuits for pain and its treatment. Sci Transl Med. 2021;13(619):eabj7360. 34757810 10.1126/scitranslmed.abj7360 PMC8675872

[11] Zhou J , Zeng F , Cheng S , et al. Modulation effects of different treatments on periaqueductal gray resting state functional connectivity in knee osteoarthritis knee pain patients. CNS Neurosci Ther. 2023. doi:10.1111/cns.14153. Advance online publication. PMC10324370 36890655

[12] Lv Q , Wu F , Gan X , et al. The involvement of descending pain inhibitory system in electroacupuncture‐induced analgesia. Front Integr Neurosci. 2019;13:38. 31496944 10.3389/fnint.2019.00038 PMC6712431

[13] Li Z , Liu M , Lan L , et al. Altered periaqueductal gray resting state functional connectivity in migraine and the modulation effect of treatment. Sci Rep. 2016;6:20298. 26839078 10.1038/srep20298 PMC4738255

[14] Sachau J , Bruckmueller H , Gierthmuhlen J , et al. The serotonin receptor 2A (HTR2A) rs6313 variant is associated with higher ongoing pain and signs of central sensitization in neuropathic pain patients. Eur J Pain. 2021;25(3):595‐611. 33171011 10.1002/ejp.1696

[15] Kang JWM , Keay KA , Kendig MD , Corbit LH , Mor D . Serotonin and dopamine show different response profiles to acute stress in the nucleus accumbens and medial prefrontal cortex of rats with neuropathic pain. Neurochem Res. 2023;48:2265‐2280. 36941432 10.1007/s11064-023-03906-y PMC10182167

[16] Alkislar I , Miller AR , Hohmann AG , et al. Inhaled cannabis suppresses chemotherapy‐induced neuropathic nociception by decoupling the raphe nucleus: a functional imaging study in rats. Biol Psychiatry Cogn Neurosci Neuroimaging. 2021;6(4):479‐489. 33622657 10.1016/j.bpsc.2020.11.015 PMC8351528

[17] Kuner R , Kuner T . Cellular circuits in the brain and their modulation in acute and chronic pain. Physiol Rev. 2021;101(1):213‐258. 32525759 10.1152/physrev.00040.2019

[18] Potewiratnanond P , le Grand SM , Srikiatkhachorn A , Supronsinchai W . Altered activity in the nucleus raphe magnus underlies cortical hyperexcitability and facilitates trigeminal nociception in a rat model of medication overuse headache. BMC Neurosci. 2019;20(1):54. 31638891 10.1186/s12868-019-0536-2 PMC6802338

[19] Shimizu S , Nakatani Y , Kurose M , et al. Modulatory effects of repeated psychophysical stress on masseter muscle nociception in the nucleus raphe magnus of rats. J Oral Sci. 2020;62(2):231‐235. 32074544 10.2334/josnusd.19-0320

[20] Gao N , Shi HP , Hu S , et al. Acupuncture enhances dorsal raphe functional connectivity in knee osteoarthritis with chronic pain. Front Neurol. 2022;12:813723. 35115998 10.3389/fneur.2021.813723 PMC8805588

[21] Lee JY , You T , Woo CW , Kim SG . Optogenetic fMRI for brain‐wide circuit analysis of sensory processing. Int J Mol Sci. 2022;23(20):12268. 36293125 10.3390/ijms232012268 PMC9602603

[22] Van Den Berge N , Albaugh DL , Salzwedel A , et al. Functional circuit mapping of striatal output nuclei using simultaneous deep brain stimulation and fMRI. Neuroimage. 2017;146:1050‐1061. 27825979 10.1016/j.neuroimage.2016.10.049 PMC5322177

[23] Tsuchiyagaito A , Misaki M , Zoubi OA , Tulsa I , Paulus M , Bodurka J . Prevent breaking bad: a proof of concept study of rebalancing the brain's rumination circuit with real‐time fMRI functional connectivity neurofeedback. Hum Brain Mapp. 2021;42(4):922‐940. 33169903 10.1002/hbm.25268 PMC7856643

[24] Han SQ , Cui Q , Guo XN , et al. Disconnectivity between the raphe nucleus and subcortical dopamine‐related regions contributes altered salience network in schizophrenia. Schizophr Res. 2020;216:382‐388. 31801675 10.1016/j.schres.2019.11.006

[25] Qin ZX , He XW , Zhang JL , et al. Altered spontaneous activity and functional connectivity in the posterior pons of patients with migraine without Aura. J Pain. 2020;21(3–4):347‐354. 31400473 10.1016/j.jpain.2019.08.001

[26] Li R , Liao W , Yu Y , et al. Differential patterns of dynamic functional connectivity variability of striato‐cortical circuitry in children with benign epilepsy with centrotemporal spikes. Hum Brain Mapp. 2018;39(3):1207‐1217. 29206330 10.1002/hbm.23910 PMC6866449

[27] Blanpied PR , Gross AR , Elliott JM , et al. Neck pain: revision 2017. J Orthop Sports Phys Ther. 2017;47(7):A1‐A83. 10.2519/jospt.2017.0302 28666405

[28] Cudkowicz ME , Shefner JM , Schoenfeld DA , et al. Trial of celecoxib in amyotrophic lateral sclerosis. Ann Neurol. 2006;60(1):22‐31. 16802291 10.1002/ana.20903

[29] Hawker K , O'Connor P , Freedman MS , et al. Rituximab in patients with primary progressive multiple sclerosis: results of a randomized double‐blind placebo‐controlled multicenter trial. Ann Neurol. 2009;66(4):460‐471. 19847908 10.1002/ana.21867

[30] LeWitt PA , Guttman M , Tetrud JW , et al. Adenosine A2A receptor antagonist istradefylline (KW‐6002) reduces "off" time in Parkinson's disease: a double‐blind, randomized, multicenter clinical trial (6002‐US‐005). Ann Neurol. 2008;63(3):295‐302. 18306243 10.1002/ana.21315

[31] Hey SP , Kimmelman J . The questionable use of unequal allocation in confirmatory trials. Neurology. 2014;82(1):77‐79. 24306005 10.1212/01.wnl.0000438226.10353.1c PMC3873626

[32] Zhao JS , Jiang S . Acupoints around the neck: a philological analysis and re‐recognition on "acupoint‐meridian" relationship. Zhongguo Zhen Jiu. 2020;40(10):1085‐1091. 33068351 10.13703/j.0255-2930.20200428-k0003

[33] Sun ZR , Yue JH , Tian HZ , Zhang QH . Acupuncture at Houxi (SI 3) acupoint for acute neck pain caused by stiff neck: study protocol for a pilot randomised controlled trial. BMJ Open. 2014;4(12):e006236. 10.1136/bmjopen-2014-006236 PMC4275681 25537784

[34] Melchart D , Streng A , Hoppe A , et al. Acupuncture in patients with tension‐type headache: randomised controlled trial. BMJ. 2005;331(7513):376‐379. 16055451 10.1136/bmj.38512.405440.8F PMC1184247

[35] Zhao L , Chen J , Li Y , et al. The long‐term effect of acupuncture for migraine prophylaxis a randomized clinical trial. JAMA Intern Med. 2017;177(4):508‐515. 28241154 10.1001/jamainternmed.2016.9378

[36] Sun M , Tao S , Geng G , et al. Identification of the optimal points for the acupuncture treatment of neck pain in China: protocol for a multicenter, matched, case‐control study. BMJ Open. 2019;9(8):e029194. 10.1136/bmjopen-2019-029194 PMC6707690 31439605

[37] Wang Y , Sun J , Zhang Z , et al. Impact of deqi on acupoint effects in patients with primary dysmenorrhea:a systematic review of randomized controlled trials. Zhongguo Zhen Jiu. 2017;37(7):791‐797. 29231558 10.13703/j.0255-2930.2017.07.027

[38] Hodges JS , Pihlstrom BL . Software support for clinical studies: review of nQuery advisor (R) release 2.0. J Dent Res. 1998;77(3):525‐526. 9496926 10.1177/00220345980770031201

[39] Driessen MT , Proper KI , Anema JR , Knol DL , Bongers PM , van der Beek AJ . Participatory ergonomics to reduce exposure to psychosocial and physical risk factors for low back pain and neck pain: results of a cluster randomised controlled trial. Occup Environ Med. 2011;68(9):674‐681. 21177661 10.1136/oem.2010.056739

[40] Wang X , Liao W , Han S , et al. Altered dynamic global signal topography in antipsychotic‐naive adolescents with early‐onset schizophrenia. Schizophr Res. 2019;208:308–316. 30772067 10.1016/j.schres.2019.01.035

[41] Braun U , Schäfer A , Bassett DS , et al. Dynamic brain network reconfiguration as a potential schizophrenia genetic risk mechanism modulated by NMDA receptor function. Proc Natl Acad Sci USA. 2016;113(44):12568‐12573. 27791105 10.1073/pnas.1608819113 PMC5098640

[42] Beliveau V , Svarer C , Frokjaer VG , Knudsen GM , Greve DN , Fisher PM . Functional connectivity of the dorsal and median raphe nuclei at rest. Neuroimage. 2015;116:187‐195. 25963733 10.1016/j.neuroimage.2015.04.065 PMC4468016

[43] Han S , He Z , Duan X , et al. Dysfunctional connectivity between raphe nucleus and subcortical regions presented opposite differences in bipolar disorder and major depressive disorder. Prog Neuropsychopharmacol Biol Psychiatry. 2019;92:76‐82. 30605709 10.1016/j.pnpbp.2018.12.017

[44] Voulgarakis P , Iakovidis P , Lytras D , Chatziprodromidou IP , Kottaras A , Apostolou T . Effects of joint mobilization versus acupuncture on pain and functional ability in people with chronic neck pain: a randomized controlled trial of comparative effectiveness. J Acupunct Meridian Stud. 2021;14(6):231‐237. 35770602 10.51507/j.jams.2021.14.6.231

[45] Blossfeldt P . Acupuncture for chronic neck pain‐‐a cohort study in an NHS pain clinic. Acupunct Med. 2004;22(3):146‐151. 15551941 10.1136/aim.22.3.146

[46] Jo HR , Noh EJ , Oh SH , et al. The effectiveness of different acupuncture therapies for neck pain A protocol for systematic review and/or network meta‐analysis. Medicine. 2021;100(16):e25379. 33879667 10.1097/MD.0000000000025379 PMC8078380

[47] Zhang F , Zhang HC . Bibliometric analysis of research trends on acupuncture for neck pain treatment over the past 20 years. J Pain Res. 2021;14:3553‐3554. 34803398 10.2147/JPR.S346284 PMC8594888

[48] Sahin N , Ozcan E , Sezen K , Karatas O , Issever H . Efficacy of acupunture in patients with chronic neck pain‐‐a randomised, sham controlled trial. Acupunct Electrother Res. 2010;35(1–2):17‐27. 20578644 10.3727/036012910803860959

[49] Bing Z , Villanueva L , Le Bars D . Acupuncture and diffuse noxious inhibitory controls: naloxone‐reversible depression of activities of trigeminal convergent neurons. Neuroscience. 1990;37(3):809‐818. 2247225 10.1016/0306-4522(90)90110-p

[50] Cao J , Tu Y , Wilson G , Orr SP , Kong J . Characterizing the analgesic effects of real and imagined acupuncture using functional and structure MRI. Neuroimage. 2020;221:117176. 32682992 10.1016/j.neuroimage.2020.117176 PMC7738388

[51] Yu S , Ortiz A , Gollub RL , Wilson G , Kong J . Acupuncture treatment modulates the connectivity of key regions of the descending pain modulation and reward Systems in Patients with chronic low Back pain. J Clin Med. 2020;9(6):1719. 32503194 10.3390/jcm9061719 PMC7356178

[52] Wei‐Yi O , Stohler CS , Herr DR . Role of the prefrontal cortex in pain processing. Mol Neurobiol. 2018;56(2):1137‐1166. 29876878 10.1007/s12035-018-1130-9 PMC6400876

[53] Trinh K , Graham N , Gross A , et al. Acupuncture for neck disorders. Spine (Phila Pa 1976). 2007;32(2):236‐243. 17224820 10.1097/01.brs.0000252100.61002.d4

[54] Yuan QL , Guo TM , Liu L , Sun F , Zhang YG . Traditional Chinese medicine for neck pain and low back pain: a systematic review and meta‐analysis. PLoS One. 2015;10(2):e0117146. 25710765 10.1371/journal.pone.0117146 PMC4339195

[55] MacPherson H , Vertosick EA , Foster NE , et al. The persistence of the effects of acupuncture after a course of treatment: a meta‐analysis of patients with chronic pain. Pain. 2017;158(5):784‐793. 27764035 10.1097/j.pain.0000000000000747 PMC5393924

[56] Zhang R , Lao L , Ren K , Berman BM . Mechanisms of acupuncture–electroacupuncture on persistent pain. Anesthesiology. 2014;120(2):482‐503. 24322588 10.1097/ALN.0000000000000101 PMC3947586

[57] Møller AR . Anatomy and physiology of pain. In: Møller AR , Langguth B , De Ridder D , Kleinjung T , eds. Textbook of Tinnitus. Springer New York; 2011:121‐132.

[58] Mertens P , Blond S , David R , Rigoard P . Anatomy, physiology and neurobiology of the nociception: a focus on low back pain (part a). Neurochirurgie. 2015;61(Suppl 1):S22‐S34. 25441598 10.1016/j.neuchi.2014.09.001

[59] Baliki MN , Chialvo DR , Geha PY , et al. Chronic pain and the emotional brain: specific brain activity associated with spontaneous fluctuations of intensity of chronic back pain. J Neurosci. 2006;26(47):12165‐12173. 17122041 10.1523/JNEUROSCI.3576-06.2006 PMC4177069

[60] Thompson JM , Neugebauer V . Amygdala plasticity and pain. Pain Res Manag. 2017;2017:8296501. 29302197 10.1155/2017/8296501 PMC5742506

[61] Coppieters I , Cagnie B , De Pauw R , Meeus M , Timmers I . Enhanced amygdala‐frontal operculum functional connectivity during rest in women with chronic neck pain: associations with impaired conditioned pain modulation. Neuroimage Clin. 2021;30:102638. 33812304 10.1016/j.nicl.2021.102638 PMC8053790

[62] Aminoff EM , Kveraga K , Bar M . The role of the parahippocampal cortex in cognition. Trends Cogn Sci. 2013;17(8):379‐390. 23850264 10.1016/j.tics.2013.06.009 PMC3786097

